# Lactobacilli-based postbiotic differentially affects chicken macrophage-like HD11 cells depending on stimulatory lipopolysaccharide dosage

**DOI:** 10.1186/s12917-025-04902-w

**Published:** 2025-07-21

**Authors:** Samuel C. G. Jansseune, Jürgen van Baal, Fany Blanc, Aart Lammers

**Affiliations:** 1 Animal Nutrition Group, Department of Animal Sciences, Wageningen University & Research, Wageningen, The Netherlands; 2 Adaptation Physiology Group, Department of Animal Sciences, Wageningen University & Research, Wageningen, The Netherlands; 3 Université Paris-Saclay, INRAE, AgroParisTech, GABI, Jouy‐en‐Josas, France; 4 Idena, Sautron, France

**Keywords:** Postbiotic, Lactobacillus, Inflammation, Macrophage, Lipopolysaccharide

## Abstract

**Background:**

This study investigated the dose-dependent effects of a lactobacilli-based postbiotic (**Post**) on the transcriptional reprogramming of the chicken macrophage-like HD11 cell line when exposed to *Escherichia coli* lipopolysaccharide (**LPS**). First, the HD11cells were treated with 0, 3, 30 and 300 ng/mL LPS in combination with 0, 0.2, 0.4, 0.6 and 0.8% v/v Post. Nitric oxide (**NO**) production was quantified at 20 h incubation and the early transcriptome reprogramming was analysed in a subset of treatments at 5 h incubation.

**Results:**

Post increased NO production dose-dependently and an LPS-postbiotic interaction was present, with the cells eliciting a higher NO production in response to Post at 30 and 300 ng/ml LPS compared to the zero and 3 ng/ml LPS. To further understand this interaction, the early transcriptome reprogramming was investigated for treatments with 0, 3 and 300 ng/mL LPS and 0 and 0.8% v/v Post. A number of differentially expressed genes were identified and gene set enrichment analysis of KEGG pathways revealed that Post at 0 and 300 ng/mL LPS influenced similar inflammation-related pathways, until Post at 3 ng/mL LPS which had a minimal effect. Expression of transcription factors (**TFs**) and immune-related genes revealed differential effects induced by Post depending on LPS concentration which would have likely influenced the inflammatory response. Specifically, the only TFs affected by Post at 300 ng/ml LPS were *STAT2*, *SMAD3* and *IFR8*, which all showed increased expression. The TFs consistently affected by Post at the zero and 3 ng/ml LPS increased and were *JUN*, *ZFP36L2*, *SMAD1* and *E2F3*.

**Conclusion:**

Our results showed that Post had a pro-inflammatory effect, which was exacerbated in the presence of a 300 but not 3 ng/ml LPS. Furthermore, the dose of LPS affected the sensitivity of the cells to Post. Dose-response studies should be performed when investigating the effects of dietary compounds on inflammation in chicken macrophages.

**Supplementary Information:**

The online version contains supplementary material available at 10.1186/s12917-025-04902-w.

## Background

Postbiotics are “a preparation of inanimate microorganisms and/or their components that confer a health benefit on the host” [1]. They attracted significant interest due to the numerous reported beneficial effects they can produce in vivo. In chickens, they were *inter alia* reported to improve performance parameters [2–4] and to modulate the immune response [2, 5, 6]. Postbiotics contain a broad range of compounds, including numerous molecules with immunomodulatory properties [7]. In a metabolomic study of a co-culture of *Lactobacillus rhamnosus* CNCM-I-3698 (**LR98**) and *L*. *formosensis* CNCM-I-3699 (**LF99**), we identified the production of molecules with immunomodulatory properties, which were also found in the derived postbiotic (**Post**) [8]. Potential compounds identified and reported for their anti-inflammatory properties in mammals included indole-3-lactic acid, 2-hydroxyisocaproic acid, genistein, lactic acid and luteolin [9–16]. Genistein has also anti-oxidative properties due to scavenging of free radicals [10]. The postbiotic from LR98 and LR99 can be expected to also contain other compounds produced by these gram-positive bacteria, such as bacterial cell fragments and exopolysaccharides [17, 18]. As reviewed by Teame et al. [19], cell fragments and exopolysaccharides of some lactobacilli strains were found to influence the inflammatory response of immune cells. Accordingly, postbiotics were reported to influence the response of innate immune cells, including macrophages [20–22].

Macrophages belong to the first immune cells involved in the defence of the host against pathogenic microorganisms and form a link with adaptive immune functions [23]. Macrophages fulfil, therefore, a central role in the immune response [24] where they are responsible for chemotaxis, elimination of pathogens, cytokine production [25, 26] and are involved in the resolution of inflammation [27]. Several lines of evidence suggest that macrophages, together with dendritic cells, continuously sense the intestinal lumen content (*e*.*g*. microbiota, pathogens, metabolites, dietary factors) [23, 28] and that their response can be modulated by multiple exogenous dietary compounds [29]. As such, immortalized macrophage-like cell lines are frequently used as a pre-screening method to analyse the immunomodulatory potential of dietary additives. (*e*.*g*. pro-, syn-, and postbiotics and phytochemicals) [30].

To assess the potential immunomodulating effect of exogenous compounds in vitro, the latter are usually tested in combination with a pro-inflammatory component of pathogenic origin. One of the best-characterized pathogen-associated molecular pattern is lipopolysaccharides (**LPS**) [31], which is highly conserved in Gram-negative bacteria and exists ubiquitously in the environment [32]. Most of the studies use a single, relatively high, dose of LPS. However, we recently reported that low (3 ng/ml) and high (300 ng/ml) effective doses of LPS induced differential transcriptomic reprogramming of HD11 chicken macrophage-like cells, suggesting that depending on the LPS dose, macrophages may be more or less sensitive to additional exogenous compounds [33]. It is, therefore, relevant to test the effects of additives in combination with different doses of LPS, something which is not commonly conducted in scientific studies. Studies using RNA-seq allow the characterisation of changes in the gene expression profile in response to external stimuli [34, 35].

This study aimed at investigating the interaction between Post and LPS at low and high dosages in chicken macrophage like cells, and to gain further insight into the underlying mechanisms. It was hypothesised and tested whether the effect of Post on the early transcriptome reprogramming of HD11 chicken macrophage-like cells differs between low (3 ng/ml) and high (300 ng/ml) effective doses of LPS used for stimulation.

## Results

### Viability

Cell viability was assessed by using an Alamar blue assay. Reduction of Alamar blue reflects the metabolic activity of the cells [36]. After 20 h stimulation, Alamar blue reduction was decreased dose-dependently by LPS from 0 to 30 ng/mL (-20 and − 38% at 3 and 30 ng/mL compared to the negative control), reaching a plateau between 30 and 300 ng/mL. Within each LPS dose, Post treatments at 0.6 and 0.8% did not affect Alamar blue reduction compared to the control.

### Nitric oxide production, iNOS and IL-12b mRNA expression

**Fig. 1:**
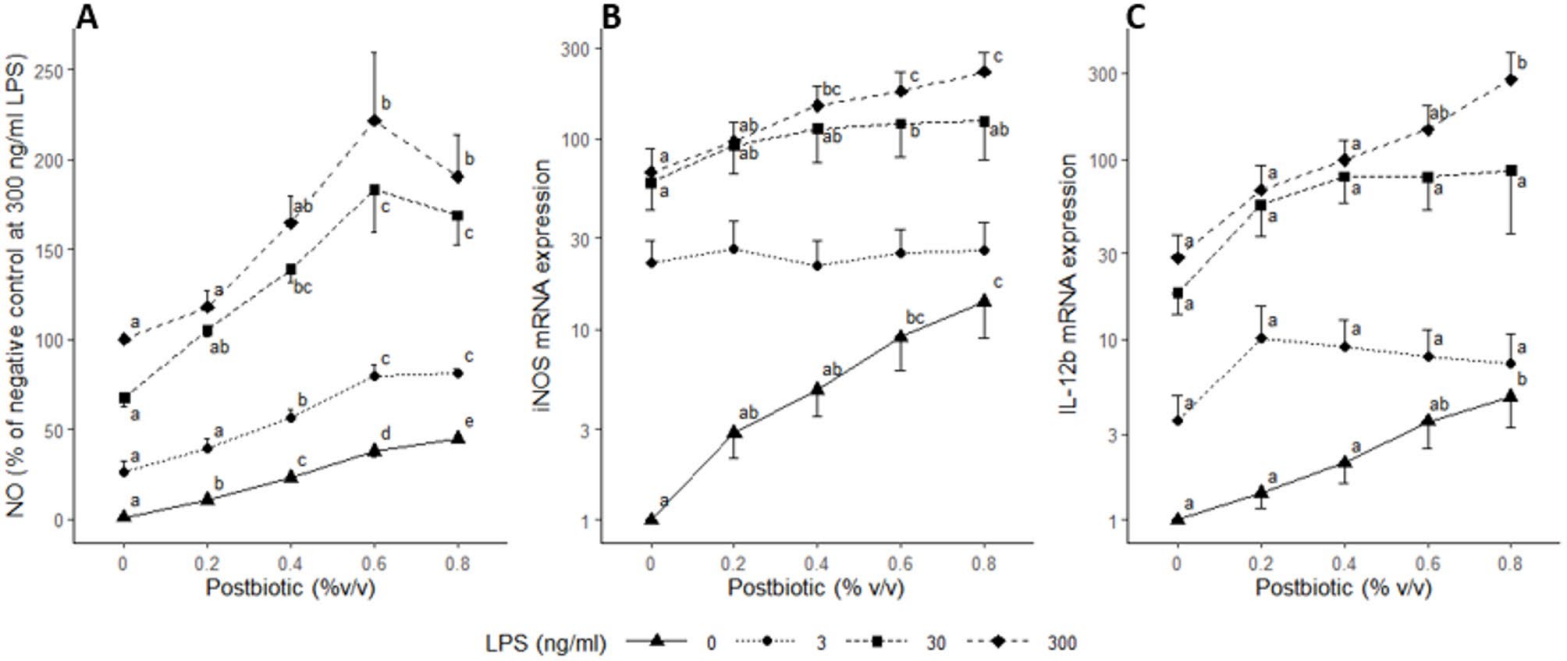
Effect of lactobacilli postbiotic on *Escherichia coli* lipopolysaccharide (LPS)-stimulated avian HD11 macrophage-like cells. Effect on nitric oxide (NO) production (**A**), and inducible nitric oxide synthase (*iNOS*) and interleukin 12b (*IL-12b*) mRNA expression (**B** and **C**, respectively). Cells were treated with medium supplemented with LPS and postbiotic. The effect of the postbiotic were analysed per LPS dose. Nitric oxide in the supernatant was quantified at 20 h incubation and values are expressed as a percentage of the 300 ng/mL LPS condition. Genes expressions were measured at 5 h incubation, are expressed relative to the negative control and are represented on a log scale. Data are represented as means ± standard error of the mean (SEM) of 5 independent experiments for NO and of 4 for *iNOS* and *IL*-12b. Means with different superscript within LPS dose are significantly different (*p* < 0.05)

Nitric oxide was measured as a marker of a proinflammatory response of HD11-cells. First, LPS was confirmed to increase NO production dose-dependently with an average of 15.0 ± 3.6 µM NO (mean ± SEM; 5 replicates of 4 wells each) at 300 ng/mL LPS. Post, in combination with all LPS doses, increased NO production in a dose-dependent manner until 0.6% v/v. Then, no significant difference was observed between the 0.6% and 0.8% v/v Post. However, for LPS at 0 ng/mL, Post continued to increase NO levels up to the highest Post concentration tested (Fig. 1). A linear regression model for Post effect including the doses from 0 to 0.6% v/v showed a significant interaction effect (*p* < 0.001) between Post and LPS concentrations. Thus, the slopes for Post effect were different depending on the LPS dose. For LPS conditions 0 and 3 and 30 and 300 ng/mL the slopes were similar (*p* = 0.87 and 0.97, respectively), but the slopes differed for LPS 0 *v*.*s*. 30, 0 *v*.*s*. 300, 3 *v*.*s*. 30 and 3 *v*.*s*. 300 ng/mL (*p* = 0.004, 0.001, 0.033 and 0.010, respectively). In macrophages, NO is produced by the enzyme nitric oxide synthetase, which is encoded by the gene inducible nitric oxide synthase (***iNOS***). Expression of *iNOS* mRNA was increased by Post at LPS concentrations of 0, 30 and 300, but not 3 ng/mL. The mRNA expression of interleukin (***IL***)*-12b* showed a dose-dependent increase by Post at 0 and 300 ng/mL LPS, but not 3 and 30 (Fig. 1).

To further investigate the dose-dependent effects of Post and LPS, a subset of six treatments were selected for transcriptomic analysis, based on NO production, *iNOS* and *IL*-12b mRNA expression for subsequent transcriptome analysis. The selected treatments were the LPS concentrations 0, 3 and 300 ng/mL and the postbiotic concentrations 0 and 0.8% v/v and their combinations.

### Transcriptional reprogramming of HD11-cells upon postbiotic and LPS stimulation

#### Sequencing and read counting

The total number of reads ranged from 34 to 81 million (Supplementary Fig. 1A). The mean quality score per sample ranged from 34.97 to 35.24 with a percentage of base ≥ 30 ranging from 88.8 to 90.1 (Supplementary Fig. 1B). The obtained sequences were then mapped to the chicken genome with an overall alignment rate of 72.4 to 76.3% (Supplementary Fig. 1C). In total 30,108 genes were mapped, out of which 10,796 remained after filtering. Those genes were used for further analysis as presented hereafter.

#### Multidimensional scaling and differential expression analysis

**Fig. 2:**
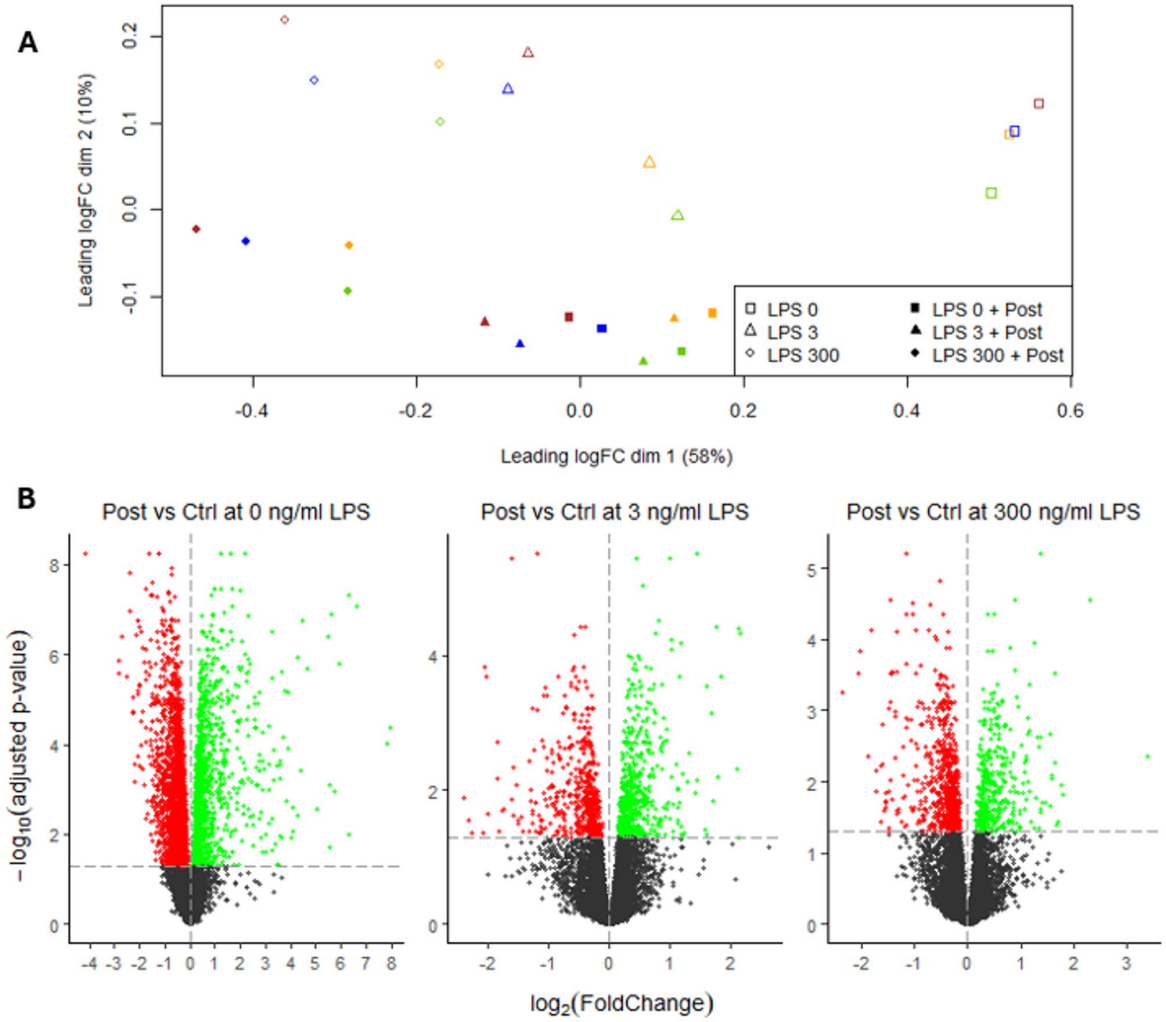
Effect of lactobacilli postbiotic on *Escherichia coli* lipopolysaccharide (LPS)-stimulated avian HD11 macrophage-like cells gene expression. (**A**) MDS-plot with one colour per replicate. (**B**) Volcano plot of the differential mRNA expression for Post effect at 0, 3 and 300 ng/mL LPS. Significant threshold was set at a false discovery rate < 0.05 (horizontal dashed line). Red, black and green dots represent upregulated, downregulated, and not significantly changed mRNA expressions, respectively

A MDS plot was performed using all genes (Fig. 2A) and revealed a good clustering of the treatment groups along the x-axis, which explained a high proportion of the variability (58%), while the postbiotic separated along the y-axis which explained 10% of the variability. The samples of the HD11 cells treated with Post and either 0 or 3 ng/mL LPS were clustered together, indicating that their expression profile is almost similar. These data suggest, therefore, that most of the variation in gene expression was due to Post but not LPS treatment, as cells treated with 3 ng LPS alone do not colocalize in the MDS plot. Then, a differential expression analysis was conducted for Post effect at each LPS concentration (Fig. 2B). In absence of LPS, the greatest number of differentially expressed genes (**DEG**) and fold change upon Post treatment were retrieved. In total, in absence of LPS, Post downregulated the expression of 2,790 genes and upregulated the expression of 1,446 genes. At 3 and 300 ng/mL LPS, Post downregulated the expression of 549 and 822 genes, respectively, while it upregulated the expression of 609 and 480 genes, respectively.

**Fig. 3:**
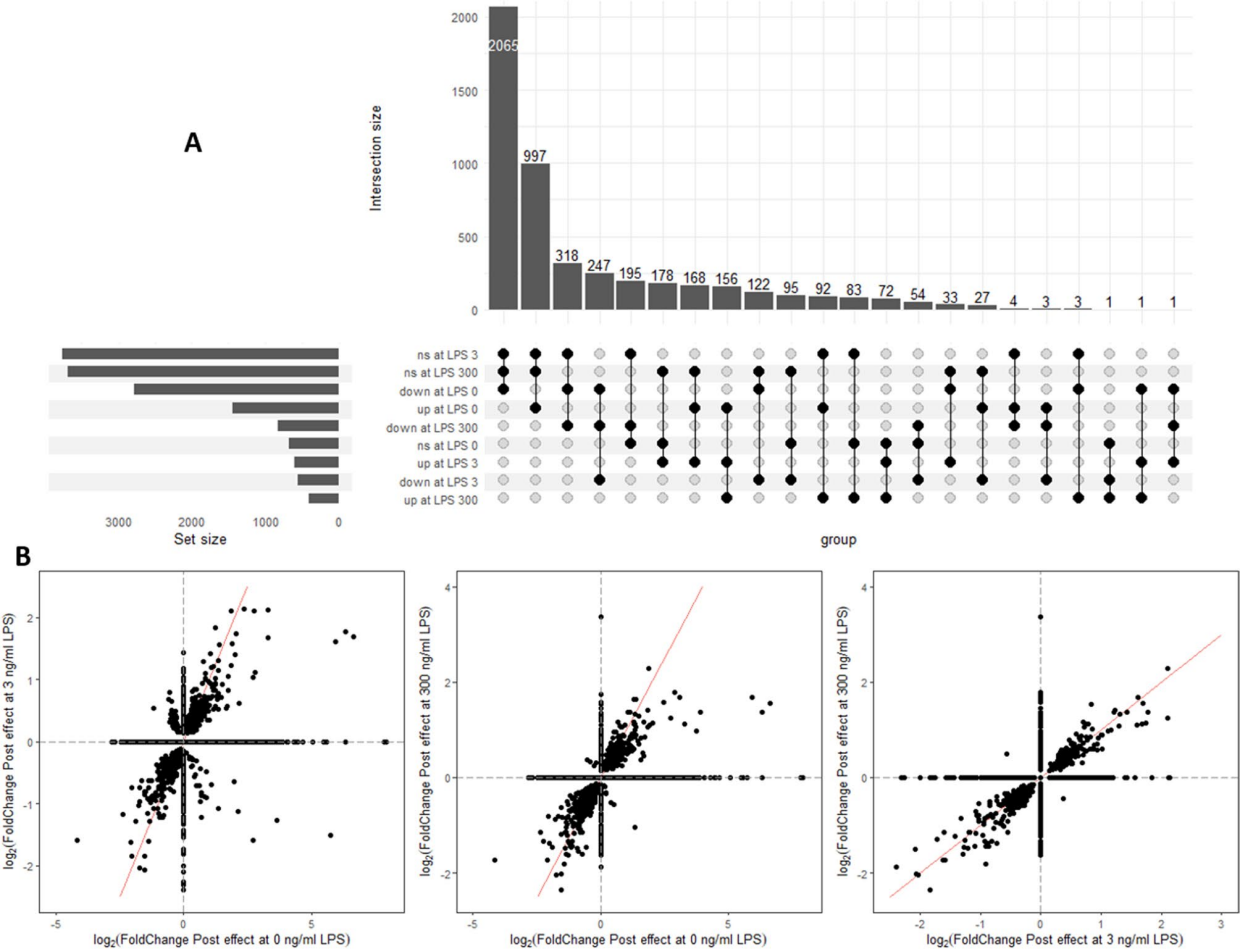
Effect of lactobacilli postbiotic on *Escherichia coli* lipopolysaccharide (LPS)-stimulated avian HD11 macrophage-like cells gene expression. (**A**) Upset plot showing among the differentially expressed genes for the effect of Post at 0, 3 and 300 ng/mL LPS, the number of genes with shared and different upregulated (up), downregulated (down), and not significantly different (ns) expression. (**B**) Correlation between log2(Fold change) for Post effect at 0, 3 and 300 ng/mL. Fold changes of non-significantly differentially expressed genes (FDR > 0.05) were set to 0 in the representation

The count of the DEG with Post in at least one of the LPS concentrations (in total 4,915 genes) are presented in an upset plot (Fig. 3A) and showed that only a minority of gene with up- and downregulated expression by Post were shared between all LPS doses, while most (91.8%) were specific for Post effect at one or two of the three LPS doses. The shared DEG with Post at all LPS doses were 247 downregulated DEG and 156 upregulated DEG. Then, the fold changes of the DEG for Post effect at 0, 3 or 300 ng/mL LPS were compared (Fig. 3B) and showed that the DEG affected by Post at 0 and 3, 0 and 300 and 3 and 300 ng/mL LPS had mainly similar fold changes.

**Fig. 4:**
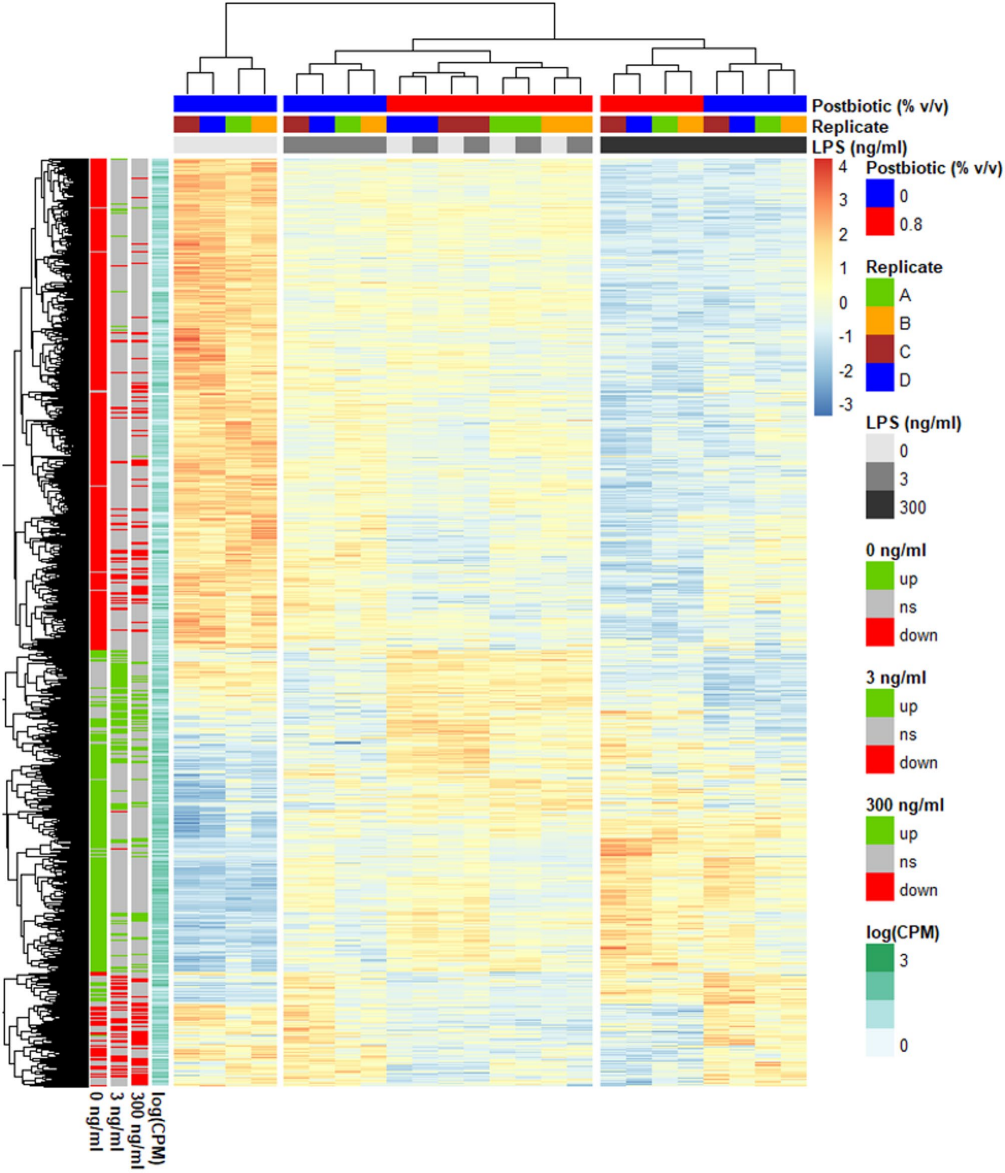
Heatmap of the mRNA gene expression profile. *Escherichia coli* lipopolysaccharide (LPS)-stimulated avian HD11 macrophage-like cells were treated with a lactobacilli postbiotic (Post). Cells were treated with 0, 3 or 300 ng/mL LPS and the effect of the postbiotic were analysed per LPS dose. Expression is normalised within each row. Only the 4915 genes with a false discovery rate < 0.05 for Post effect in at least one LPS concentration are represented. Per LPS dose, ns stands for non-significant effect of the postbiotic, up for upregulation and down for downregulation of the gene expression by the postbiotic. CPM: count per million

Then, the DEG with Post in at least one of the three LPS doses were plotted in a heatmap (Fig. 4) together with a hierarchical clustering dendrogram on gene expression and samples showing which genes and samples had a greater similarity in expression pattern. In absence of LPS, Post induced a transcriptomic profile close to the one of Post with 3 ng/mL LPS, as shown by the samples clustering per replicate but not per treatment. Stimulation with 3 ng/ml LPS, compared to no LPS, had a minor influence on the effect of Post on the transcriptomic profile. At the highest dose of LPS, the cells treated with and without the postbiotic clustered separately per treatment indicating a substantial change induced by Post.

#### Gene set enrichment analysis of KEGG pathways

**Fig. 5:**
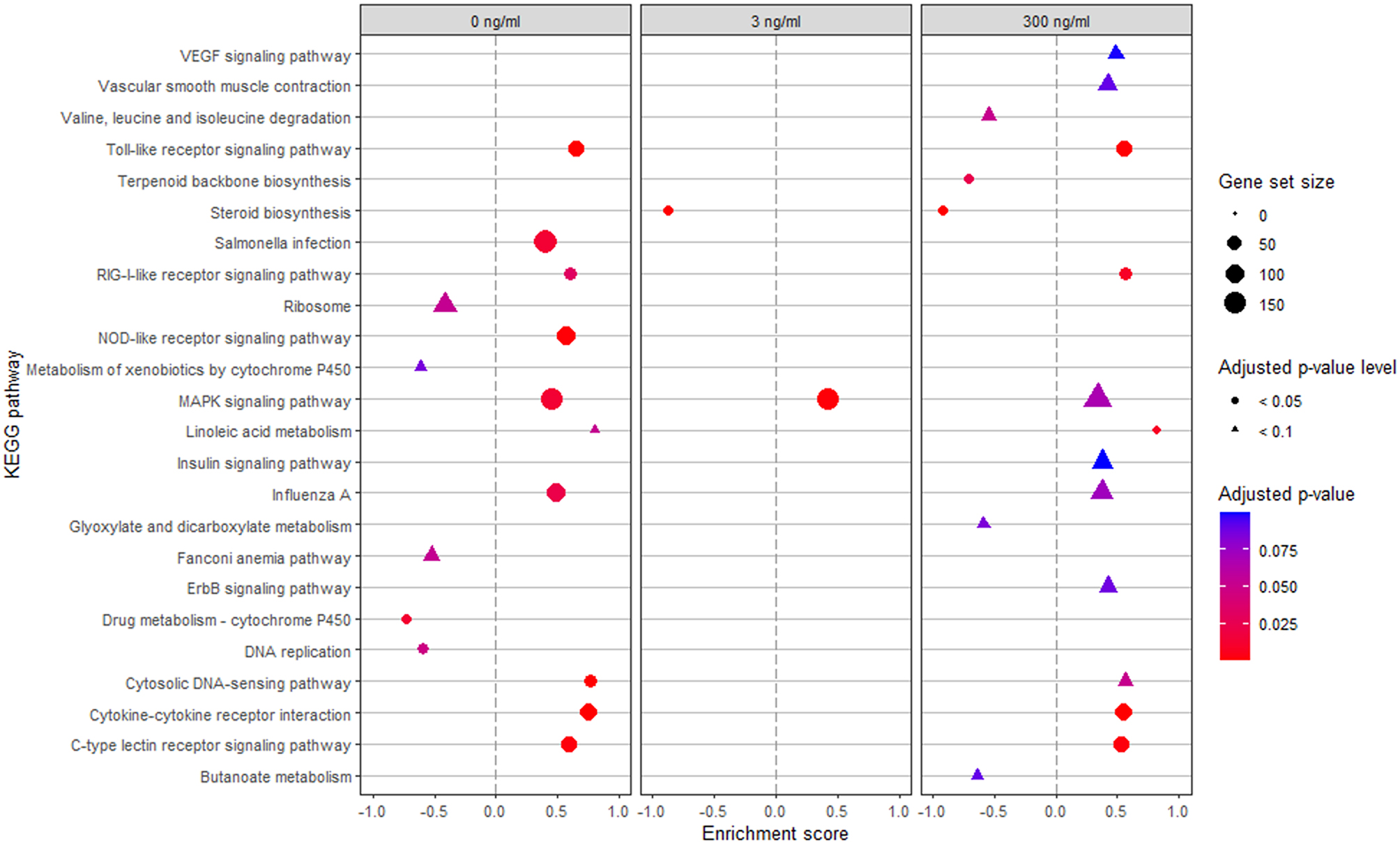
Gene set enrichment analysis of KEGG pathways. *Escherichia coli* lipopolysaccharide (LPS)-stimulated avian HD11 macrophage-like cells were treated with a lactobacilli postbiotic (Post). Cells were treated with 0, 3 or 300 ng/mL LPS and the effect of the postbiotic were analysed per LPS dose. Only pathways with an adjusted-*p* < 0.1 for Post effect are presented

To further clarify the potential functional roles of the DEG, a gene set enrichment analysis of KEGG pathways was performed. Pathways with an adjusted-*p* < 0.1 are presented in Fig. 5. Only the MAPK signalling pathway was enriched by Post across all LPS conditions. Seven pathways were enriched by Post at both 0 and 300 ng/mL LPS, namely the toll-like, RIG-I-like, and C-type lectin receptor signalling pathways, linoleic acid metabolism, Influenza A, and Cytosolic DNA-sensing pathways. Notably, no pathways with reduced enrichment scores due to Post were consistently found for all LPS conditions, but steroid biosynthesis showed reduced enrichment at both 3 and 300 ng/mL LPS.

#### Expression of immune genes

To examine the response of the macrophages, we identified all genes that were associated with Gene Ontology:0002376 “immune system process” as well as the one related to KEGG immune system subcategory 04620, 04621, 04622, 04623, 04625 and 04672, KEGG membrane transport subcategory 02010, KEGG signal transduction subcategory 04010, 04020, 04060, 04350 and 04370, KEGG infectious disease subcategory 05132, 05164 and 05168, KEGG cellular processes subcategory 04115, 04218, 04110, 04142, 04145 and 04146. Of these genes, the transcription factors (**TFs**) and co-TFs, as identified from AnimalTFDB v4.0 (Shen et al., 2022) were separated from the other genes.

**Fig. 6:**
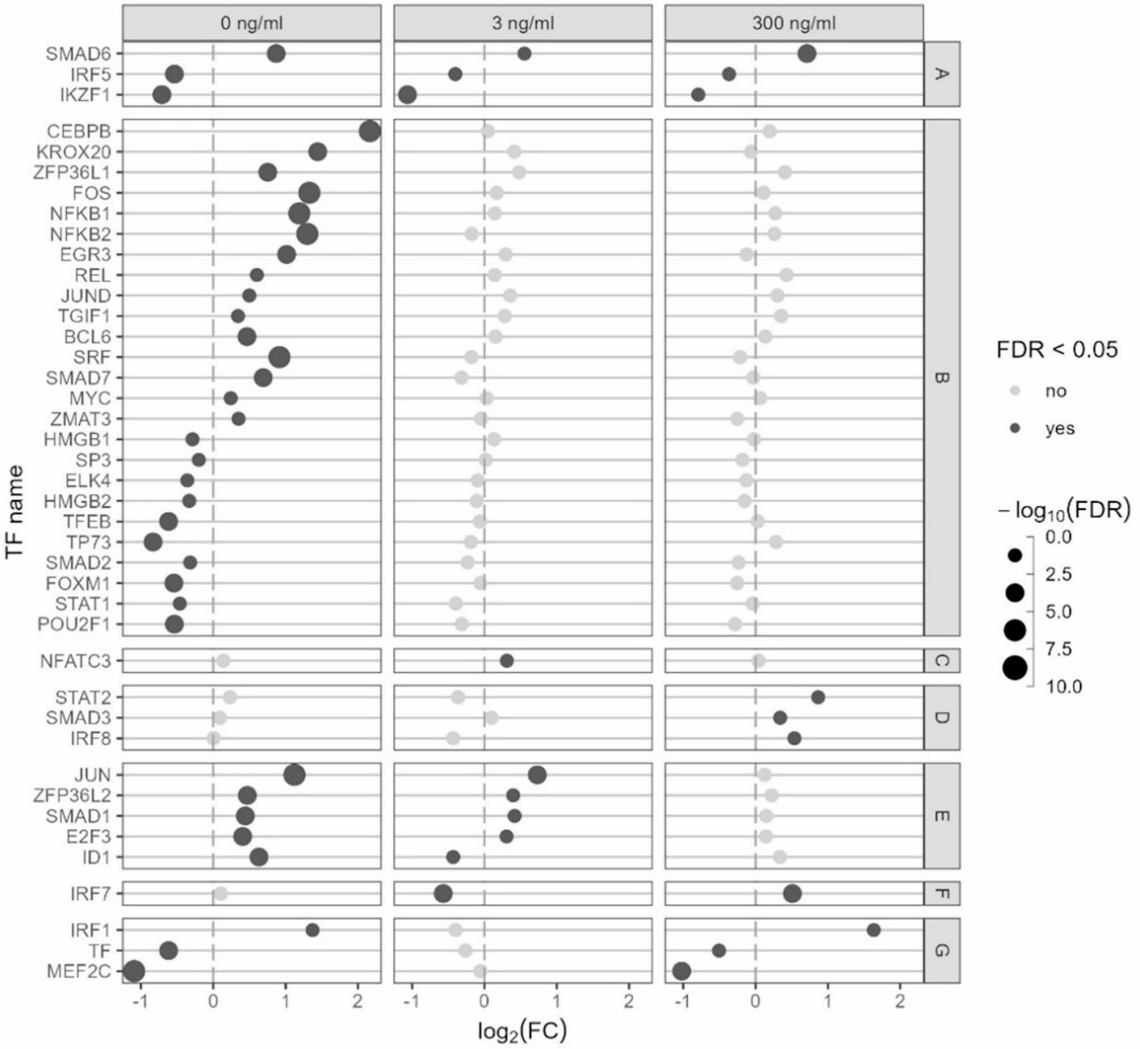
mRNA expression of immune related transcription factors (TFs). *Escherichia coli* lipopolysaccharide (LPS)-stimulated avian HD11 macrophage-like cells were treated with a lactobacilli postbiotic (Post). Cells were treated with 0, 3 or 300 ng/mL LPS and the effect of the postbiotic were analysed per LPS dose. Represented TFs have a false discovery rate < 0.05 in at least one comparison. TFs were separated in seven categories depending on Post effect at the different LPS doses; **A:** affected at all LPS doses, **B:** affected at 0 ng/mL LPS only, **C:** affected at 3 ng/mL LPS only, **D:** affected at 300 ng/mL LPS only; **E:** affected at 0 and 3 ng/mL LPS; **F:** affected at 3 and 300 ng/mL LPS and **G:** affected at 0 and 300 ng/mL LPS

To further elucidate the expression pattern of the TFs in response to Post, the TFs with a false discovery rate (**FDR**) < 0.05 for Post effect in at least one LPS dose were separated into seven categories (A to G) and represented in Fig. 6. Only three TFs were affected in the same direction by Post at all LPS concentrations (A): *SMAD6* which increased and interferon regulatory factor (***IRF***) *5* and *IKZF1*, which decreased. Twenty-five TF were only affected by Post in absence of LPS, with the most affected ones being *CEBPB*, *KROX20*, *FOS*, *NF-kB1* and *NF-kB2* (B). Only *NFATC3* was affected by Post at 3 ng/mL LPS only (C). Three TFs were only affected at 300 ng/mL LPS: *STAT2*, *SMAD3* and *IRF8* (D). Five TFs were affected at both 0 and 3 ng/mL LPS (E). One TF was affected at 3 and 300 ng/mL but in an opposite way (F). Finally, three TFs were affected only at 0 and 300 ng/mL LPS: *IRF1* which increased, and *TF* and *MEF2C* which decreased (G).

**Fig. 7:**
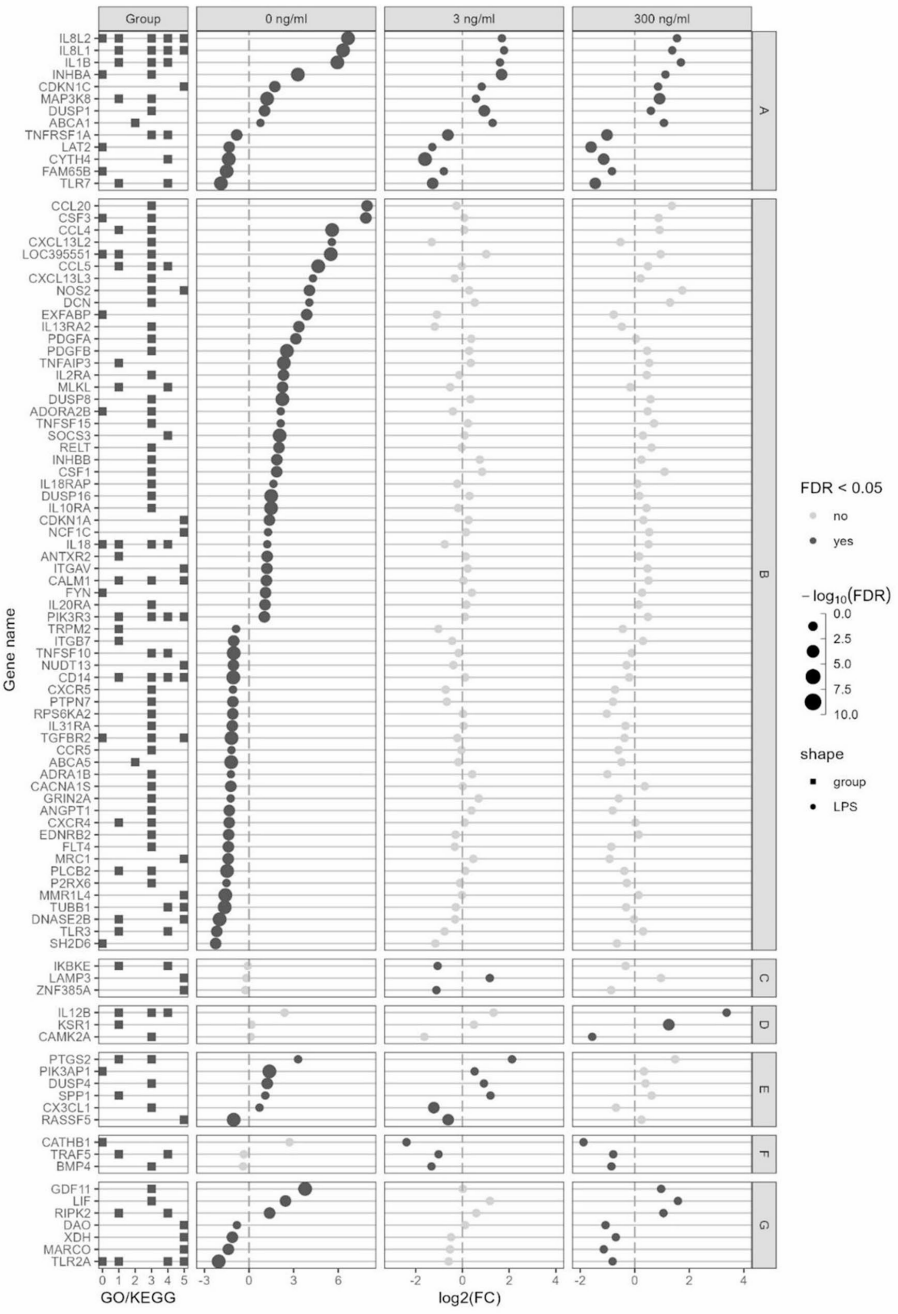
mRNA expression of immune related genes. *Escherichia coli* lipopolysaccharide (LPS)-stimulated avian HD11 macrophage-like cells were treated with a lactobacilli postbiotic (Post). Cells were treated with 0, 3 or 300 ng/mL LPS and the effect of Post were analysed per LPS dose. Represented genes have false discovery rate < 0.05 and a|log2(fold change)| > 1 in at least one comparison. Gene were separated in seven categories depending on Post effect at the different LPS doses; **A:** affected at all LPS doses, **B:** affected at 0 ng/mL LPS only, **C:** affected at 3 ng/mL LPS only, **D:** affected at 300 ng/mL LPS only; **E:** affected at 0 and 3 ng/mL LPS; **F:** affected at 3 and 300 ng/mL LPS and **G:** affected at 0 and 300 ng/mL LPS. Group GO/KEGG: 0: GO:0002376 immune system process; 1: KEGG immune system 04620, 04621, 04622, 04623, 04625 and 04672; 2: KEGG membrane transport 02010; 3: KEGG signal transduction 04010, 04020, 04060, 04350 and 04370; 4: KEGG infectious disease 05132, 05164 and 05168; 5: KEGG cellular processes 04115, 04218, 04110, 04142, 04145 and 04146

For immune-related genes other than TFs, the ones with a FDR < 0.05 and a|log_2_(fold change)| > 1 for Post effect in at least one LPS dose were represented in Fig. 7. Those genes were clustered in seven categories depending on the effect of Post: affected at all LPS doses (A; 8 upregulated and 4 downregulated), at 0 (B; 35 upregulated and 27 downregulated), 3 (C; 1 upregulated and 2 downregulated) and 300 (D; 2 upregulated and 1 downregulated) ng/mL only, at 0 and 3 (E; 4 upregulated and 1 downregulated; 1 upregulated at 0 but downregulated at 3 ng/mL), 3 and 300 (F; 3 downregulated), and 0 and 300 (G; 3 upregulated and 3 downregulated) ng/mL.

## Discussion

Macrophages are key cells of the chicken immune system, contributing to the protection of the host against infection. Immortalized macrophage-like cell lines are often used as a convenient model to pre-screen the immunomodulatory potential of dietary compounds (*e*.*g*. pro-, syn- and postbiotics and phytochemicals) in vitro for use as feed additives (reviewed in Paradowska et al. [30]). They are commonly tested using a single, usually high, dose of LPS to determine if the compounds could interfere with the LPS-induced inflammatory response. However, we recently reported that low (3 ng/ml) and high (300 ng/ml) effective doses of LPS caused differences in the transcriptomic profile of HD11 macrophage-like cells, indicating that depending on the LPS dose, macrophages may be more or less sensitive to additional exogenous compounds [33]. Here we investigated the dose-effect of Post on the immune response of chicken macrophages under various doses of LPS-stimulation and performed transcriptomic analysis to gain more insight into the involved biological mechanisms.

Measurement of NO production clearly showed an LPS-Post interaction, with the pro-inflammatory effect of Post being more pronounced at 30 and 300 ng/ml LPS compared to zero and 3 ng/ml LPS. This interaction was confirmed by a different effect on the early transcriptome reprogramming by Post depending on the LPS doses confirming the hypothesis that the effect of Post differs between low (3 ng/ml) and high (300 ng/ml) doses of LPS used for stimulation. A dose-dependent increase in *iNOS* expression was observed, except at 3 ng/ml LPS. The time difference in the analysis of NO and iNOS (20 h vs. 5 h) could explain the different pattern in response to Post. The increase of NO at 3 ng/ml LPS may have resulted from other post-transcriptional regulations (e.g. enzyme stability and activity). Accordingly, at 3 ng/ml LPS, Post had a limited effect on the transcriptomic profile of the cells. Despite Post was shown to contain anti-inflammatory components [8], a pro-inflammatory effect was observed. This suggests that pro-inflammatory components were present in Post which could be due to specific metabolites in Post or originate from cell wall components of the lactobacilli. Accordingly, lactobacilli peptidoglycan or teichoic acids were reported to affect the response of dendritic cells and macrophages [37–39].

Concurrently with the increase in NO production, at all LPS doses, Post increased the expression of the pro-inflammatory genes *IL-1b*, and the two *IL-8* subunits *L1* and *L2*, which are characteristic of classically activated macrophages (M1 macrophages). With regards to the effect of Post independently of the LPS concentration, the mRNA expressions of three TFs were affected. *SMAD6* increased, while the *IRF5* and *IKZF1* decreased. In mice, SMAD6 was reported to be a mediator of the anti-inflammatory signal in macrophages promoting M2 polarization [40, 41]. IRF5 is a marker of classically activated (M1) macrophages [42–44]. The *IKZF1* mRNA encodes the Ikaros TF which was reported to repress IRF5 expression in human macrophages [45], but Ikaros was also reported to play a key role in maintaining the NF-kB pro-inflammatory role in a dose-dependent manner [46]. The differential expression of these TFs may indicate that the Post-induced pro-inflammatory response, in terms of transcriptomic reprogramming, may have peaked before 5-hour post-stimulation, and that by this time, the cells had already transitioned into a regulatory state. Another explanation is that the presence of primarily anti-inflammatory metabolites in Post [47] activated some anti-inflammatory pathways, but that the latter were not sufficient to counteract the effects of pro-inflammatory components in Post. Consistently with the upregulation of M1 inflammatory factors, at 0 and 300 ng/ml LPS, Post induced the enrichment of immune-related KEGG pathways: namely Toll-like, MAPK and C-type lectin receptor signalling, cytosolic DNA-sensing and the cytokine-cytokine receptor interaction pathways. The upregulation of these pathways indicates that Post has a substantial pro-inflammatory effect on HD11 cells, rather than the expected anti-inflammatory effect, as suggested by the presence of anti-inflammatory metabolites.

The chemokine ligand 20 (***CCL20***) and the colony-stimulating factor 3 (***CSF3***) were the DEG with highest fold change with Post treatment only. These factors have a clear function with respect to the immune response of cells. The inflammatory chemokine CCL20 is multifunctional and plays a role as a chemoattractant for immune cells in case of immune stimulation, but also participates in maintaining immune homeostasis and inflammatory response through T helpers lymphocytes pathways [48]. The *CSF3* gene encodes the granulocyte colony-stimulating factor (**G-CSF**) which is key in the proliferation, differentiation and progression of neutrophil cell lineages [49]. The molecule G-CSF is mainly produced by monocytes and macrophages [49]. The *CSF3* gene may play a role to sustain iNOS expression [50] and could, therefore, have supported the observed increased of *iNOS* mRNA expression by Post. Regarding TFs, the one with highest fold change with Post in absence of LPS was CCAAT enhancer binding protein beta (***CEBPB***). The latter was also the TF with the highest increase due to LPS stimulation [33]. Zimmermann et al. [51]. reported that LPS increased the activity of C/EBPβ, the enzyme encoded by the *CEBPB* mRNA, in HD11 macrophages, promoting the pro-inflammatory response. The C/EBPβ protein was also reported to promote inflammation in LPS-stimulated mammalian macrophages [52]. Therefore, the fact that *CEBP* expression is increased by Post provides extra evidence that Post has a pro-inflammatory effect on macrophages.

Among the TFs potentially responsible for the differential effect of Post depending on the LPS concentration are the TFs being increased or decreased by Post only at 300 ng/ml LPS or at both the null and 3 ng/ml LPS doses. Among the TFs being affected by post at 300 ng/ml LPS only are *SMAD3*, the signal transducer and activator of transcription 2 (***STAT2***), and *IFR8*. The SMAD3 was reported to promote anti-inflammatory macrophages [53, 54]. Similarly, the STAT2 signalling was reported to repress macrophage activation in mice [55]. The upregulated expression of those two anti-inflammatory TFs tends to indicate that the cells were regulating their pro-inflammatory activation, potentially to avoid an over-reaction, but further research is required to investigate this regulation. The IRF8 plays a complex role in macrophages, depending on the status of the cells [56]. Increased mRNA expression of *IRF8* in response to LPS stimulation was also reported in mouse macrophages [56, 57], and was reviewed to promote the transcription of pro-inflammatory cytokines, including IL-12 [57], as observed in our study. The TFs affected by Post at the null and 3 ng/ml LPS doses are *JUN*, *ZFP36L2*, *SMAD1* and *E2F3*. *SMAD1* is activated by the anti-inflammatory cytokine TGF-β1 in human macrophages [58]. The protein ZFP36L2 was reported to suppress the function of regulatory T cells [59], therefore suggesting a pro-inflammatory effect of this protein. In chicken HD11 cells, up-regulation of JUN was reported to contribute to the stimulation of the immune response against *Salmonella typhimurium* endotoxin [24]. GDF11 was reviewed to be involved in the regulation of inflammation in mammals, mainly by acting as an anti-inflammatory agent, but the exact implication of GDF11 remains to be investigated [60, 61]. E2F TFs were reported for their role in promoting inflammation and multiplication in cancer cells [62]. Activation of different TFs by Post depending on the dose of LPS may have likely contributed to the observed Post-LPS interaction effect. However, the characterization of their exact role in chickens is, for most TFs, too limited to further explain the differential effect of Post at the different LPS doses.

In the absence of LPS, Post up- and downregulated the expression of more immune-related genes and TFs than at low (3 ng/ml) and high (300 ng/ml) LPS doses. Furthermore, the expression of numerous immune-related genes and TFs were differentially affected between 3 and 300 ng/ml LPS. Whether these differences are the result of a different response of the cells, or a time-related effect remains to be investigated. Nevertheless, our results indicate that depending on the dose of LPS at which co-stimulation with Post was applied, different cellular pathways were involved. This indicates that when using macrophage-like cell lines as a model to study dietary interventions, the design must include a range of LPS doses to avoid misinterpretation of the cellular pathways activated by the dietary ingredient.

Bacterial derived dietary supplements can influence the chicken immune response. In vitro studies on macrophages contribute to understand such effects [63]. Here, we used the macrophage cell line to investigate whether Post could have an immunomodulatory effect. The latter was observed to depend on the concentration of antigens present in the medium. Specifically, our results suggest that Post may interact with macrophages to increase their pro-inflammatory response, particularly when in contact with high concentrations of antigens, despite the mechanisms underlying our observations are not fully understood. If occurring in vivo, these mechanisms may contribute to the protection of the host by inducing a stronger immune response against pathogens. Considering the complexity of the gut environment, potential effects of digestion on Post, altering its composition and the fact that only the liquid fraction was studied, dedicated studies are required to confirm if Post can modulate the immune response in vivo.

## Conclusions

Post increased inflammatory markers in chicken macrophage-like HD11 cell line stimulated with LPS. The early transcriptome reprogramming was dependent on the LPS dose used for stimulation. This indicates that components tested using LPS for stimulation of in HD11 cells can have different effects depending on the dose of LPS. Moreover, Post treatment resulted in fewer differentially expressed TF and immune-related genes with LPS stimulation than without LPS. This study showed for the first time the dose-dependent interaction of a postbiotic with LPS on chicken macrophage-like cell responses, and demonstrates the usefulness of RNA-seq combined with pathways analyses to understand the effects of this interaction. Studies in the mode of action of dietary components using in vitro models should investigate varying dosages of stimulatory agents.

## Materials and methods

### Cell culture and subculture

The chicken macrophage-like cell line HD11 constitutes an established chicken myelomonocytic cell line transformed by MC29 virus [64]. The HD11 cells, kindly provided by Dr. C. A. Jansen of the Cell and Immunology Group of Wageningen University (Wageningen, the Netherland), were maintained in Roswell Park Memorial Institute (**RPMI**) 1640 containing GlutaMAX, phenol red and 25 mM HEPES (Gibco, Thermo Fisher Scientific, Waltham, USA) and supplemented with 8% v/v heat inactivated foetal calf serum (Gibco) and 1% v/v Penicillin-Streptomycin 100 U/mL (Gibco). HD11 cells were always incubated at 41 °C under 5% CO2 in a humidified atmosphere. Cells were subcultured twice a week by preliminary washing with prewarmed (41 °C) Dulbecco’s Phosphate Buffer Saline without calcium and magnesium (**DPBS**) (Gibco) followed by two 5 min washes with DPBS supplemented with 0.5 mM EDTA. The cell suspension was centrifuged (200 × g; 5 min; 22 °C) to remove EDTA before reseeding the cells. Prior to reseeding, the cells were counted in a counting chamber (Optik-Labor, Görlitz, Germany) and resuspended in the appropriate volume of culture medium. For the experiments, cells at 80–90% confluency were subcultured in 96-well-culture plates at 2 × 10^5^ cells in 200 µL per well (Cellstar, Greiner Bio-One, Kremsmünster, Austria). After overnight incubation, medium was replaced by an equal volume of treatment solutions.

### Cells stimulation

Postbiotic from LR98 and LF99 (**Post**) was purchased from STI biotechnologie (Maen Roch, France) and stored at -70 °C. For use, the postbiotic solution was centrifuged (16,000 × g, 30 min, 25 °C) prior to 0.22 μm filtration of the liquid fraction. The LPS solution at 1 mg/mL was prepared by diluting purified LPS derived from *Escherichia coli* (serotype O55:B5, cat. no. L2880; Sigma Chemical Co. St. Louis, MO, USA) in RPMI 1640 and stored at -20 °C before use. HD11 cells were treated with 0, 0.2, 0.4, 0.6 and 0.8% v/v postbiotic solution in combination with 0, 3, 30 and 300 ng/mL LPS solution, prepared by dilution in RPMI 1640 containing GlutaMAX, phenol red and 25 mM HEPES (Gibco) and supplemented with 1% v/v penicillin-streptomycin at 100 U/mL both (Gibco). The treatments were present on four wells per microplates and the experiment were replicated five times.

### Measurement of nitric oxide production

Per well, the nitric oxide (**NO**) production of the cells was determined indirectly, after 20 h of incubation, by measuring the production of the reactive nitrogen intermediate nitrite, with the Griess colorimetric assay [65]. Briefly, 50 µL cell supernatant was mixed with an equal volume of Griess reagent and incubated for 10 min at room temperature. Griess reagent was obtained by mixing a solution of 0.2% w/v naphthylethylenediamine dihydrochloride in MilliQ water with a solution of 2% w/v sulfanylamide in 5% H_3_PO_4_. Absorbance was read at 540 nm in a Multiskan GO (Thermo Fisher Scientific) and nitrite concentration was determined from a nitrite calibration curve ranging from 0 to 100 µmol.

### Measurement of cell viability

Per well, the viability of stimulated HD11 cells was evaluated using Alamar blue, a non-toxic cell-permeable solution [36]. Briefly, after 20 h incubation, the medium in the culture plate was emptied and immediately replaced by 100 µL of a diluted Alamar blue solution (Resazurin Sodium Salt at 0.4 g/mL). The test was performed for all LPS conditions (0, 3, 30 and 300 ng/mL) in combination with 0, 0.6 and 0.8% v/v Post. A stock solution of Alamar blue was made by dissolving 1 g of Resazurin Sodium Salt (BioReagent, R7017, Sigma-Aldrich Chemie GmbH, Schnelldorf, Germany) in 100 mL sterile phosphate-buffered saline (Gibco) followed by sterile filtering through a 0.22 μm pore size syringe filter (Acrodisc, Pall Laboratory Corporation, New York, NY, USA) and stored at -20 °C protected from light until further use. For use, the stock solution was diluted 250 times in prewarmed culture medium without foetal calf serum. Cells with Alamar blue solution were incubated at 41 °C in 5% CO_2_ and 95% humidity for 50 min and protected from light. Incubation time was determined empirically to be adapted to the conditions of the current experiment (cell density and activity) and to allow the detection of differences in Alamar blue reduction between treatments. Reduction of Alamar blue, the marker of cell viability was quantified by measuring fluorescence at 590 nm following excitation at 560 nm (Multiscan GO, Thermo Fisher Scientific), as presented by Zachari et al. [66].

### Gene expression analysis

Samples for gene expression analyses were collected after 5 h stimulation. Cells were lysed with RA1 buffer, the content of the four wells per treatment replicate were combined, and RNA was isolated using the RNA minikit (Thermo Fisher Scientific). RNA quantity and quality were checked by Nanodrop (ND-1000; ThermoScientific). Subsequently, 500 ng total RNA was reverse-transcribed to cDNA with Superscript III (Thermo Fisher Scientific) and T1 thermocycler (Biometra, Göttingen, Germany) for 5 min at 25 °C followed by 60 min at 50 °C, 15 min at 55 °C, 15 min at 70 °C and kept at 4 °C until storage at -30 °C. 1/50 diluted cDNA aliquots were quantified with QuantStudio 5 (Thermo Fisher Scientific) and SensiFast SYBR Lo-ROX kit (Bioline, Paris, France). Briefly, 1 µL of each primer (5 µM), 10 µL SensiFast mix and 4 µL water were added to 5 µL of cDNA sample. The used primer sequences are presented in Table 1. The PCR protocol was: 2 min holding at 95 °C followed by 35 to 40 cycles of 15 s at 95 °C and 30 s at 60 °C. The PCR was followed by melting curve analysis to confirm specific amplification of its target mRNA. The iNOS and IL-12b mRNA expression were calculated using the 2^-∆∆CT^ method [67] and glyceraldehyde-3-phosphate dehydrogenase, ribosomal protein lateral stalk subunit P0 and peptidylprolyl isomerase A as housekeeping genes.

**Table 1:** Primers used for RT-qPCR

Target^1^	Forward 5’-3’	Reverse 5’-3’
*GADPH*	ATCCCTGAGCTGAATGGGAAG	AGCAGCCTTCACTACCCTCT
*RPLP0*	TTGGGCATCACCACAAAGATT	CCCACTTTGTCTCCGGTCTTAA
*PPIA*	CCCGTCGTGTTCTTCGACAT	CCCTTGTAGCCAAATCCCTTCT
*IL-12b*	CCCAGATGCTGGCAACTACA	GAACGTCTTGCTTGGCTCTTT
*iNOS*	CTACCAGGTGGATGCATGGAA	ATGACGCCAAGAGTACAGCC

^1^*GAPDH*: Glyceraldehyde-3-phosphate dehydrogenase; *RPLP0*: Ribosomal protein lateral stalk subunit P0; *PPIA*: Peptidylprolyl isomerase A; *IL*: Interleukin; *iNOS*: Inducible nitric oxide synthase

### Transcriptome analysis

For transcriptome analysis, a subset of samples of different treatments was selected based on NO production. For each selected treatment group, 4 replicates were analysed. First, RNA quantity and quality were confirmed by using the Qubit (Thermo Fisher Scientific) and the Bioanalyzer (Agilent, Santa Clara, USA), respectively. Then, samples were sent to GENEWIZ/Azenta (Leipzig, Germany) for RNAseq. Briefly, PolyA selection was performed via NEBNext Poly (A) mRNA Magnetic Isolation Module (New England Biolabs, Ipswich, USA). Then, the NEBNext Ultra II RNA Library Preparation Kit (New England Biolabs) was used according to manufacturer protocol, and samples were sequenced with a NovaSeq 6000 (Illumina, San Diego, USA) with the 2 × 150 bp configuration and ~ 30 M pair-ends reads per sample.

The preprocess of RNAseq data was performed with Galaxy [68]. First, data were trimmed with Sickle v1.22.2 with default parameters. Then, reads were aligned to the *Gallus gallus* bGalGal1.mat.broiler.GRCg7b reference genome and version 110 for known spliced sites (Ensembl) with HISAT2 v.2.2.1 using default parameters except for intron length (set to 10,000). Aligned reads were counted with htseq-count v.0.9.1 with union mode for overlapping reads and other parameters as default. Among the list of genes detected by RNA-seq, the TFs and co-TFs were separately identified using AnimalTFDB v4.0 [69].

### Statistical analyses

Data analysis was performed with R v.4.3.1 [70]. The differences were considered significant at *p*-values or adjusted-p less than 0.05.

The effects of Post on nitric oxide, viability and gene expression data were analysed by ANOVA, with Post as a factor effect and a random replicate effect, followed by a Tuckey posthoc test. To assess the effect of Post on NO production at the different LPS concentrations, an ANCOVA was performed with LPS as a factor, Post as a continuous variable, their interaction and a random replicate effect, followed by a Tuckey posthoc test for Post effects.

For the transcriptome, differential expression analysis was performed with edgeR v.3.42.4 [71]. First, the low-expressed genes were filtered with filterByExpr and the genes with an ambiguous NCBI gene annotation were removed. Then, library sizes were normalised per treatment group with normLibSize and the negative binomial generalized model was built with glmQLFit. Comparisons between treatments for fold change and *p*-values were performed with glmQLFTest and genes with false discovery rate < 0.05 were considered to be significantly differentially expressed. The multidimensional scaling (**MDS**) plot was built with plotMDS from edgeR. Kyoto Encyclopedia of Genes and Genome (**KEGG**) pathways gene set enrichment analysis between treatments was performed on the complete gene list ordered by fold change with gseKEGG from package clusterProviler v.4.8.2 [72]. using the *Gallus gallus* reference, a *p*-value cut-off of 0.05 and a seed. Then, the significantly affected pathways were individually represented with pathview, while plotting only the differentially expressed genes.

## Electronic supplementary material

Below is the link to the electronic supplementary material.

**Supplementary Material 1**: **Supplementary Fig. 1**. Total reads number (A), reads quality (B) and overall mapping rate (C) of RNA-seq data. On B, each dot represent a sample.

## Data Availability

The datasets used and analysed during the current study are available from the corresponding author on reasonable request. Sequence data that support the findings of this study have been deposited in the NCBI Sequence Read Archive (SRA) database under the Bioproject accession number PRJNA1165447.
