# Supplementary figures and images for: Lactobacilli-based postbiotic differentially affects chicken macrophage-like HD11 cells depending on stimulatory lipopolysaccharide dosage
Source: BMC Vet Res. 2025 Jul 21;21:479. doi: 10.1186/s12917-025-04902-w (PMC12278625; doi:10.1186/s12917-025-04902-w)

A

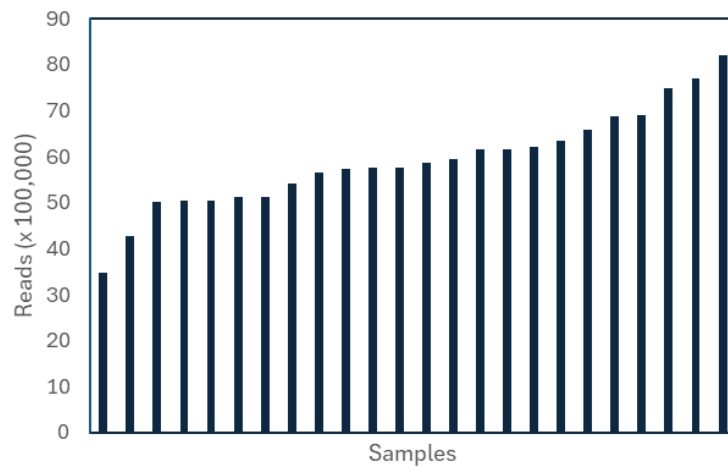

B

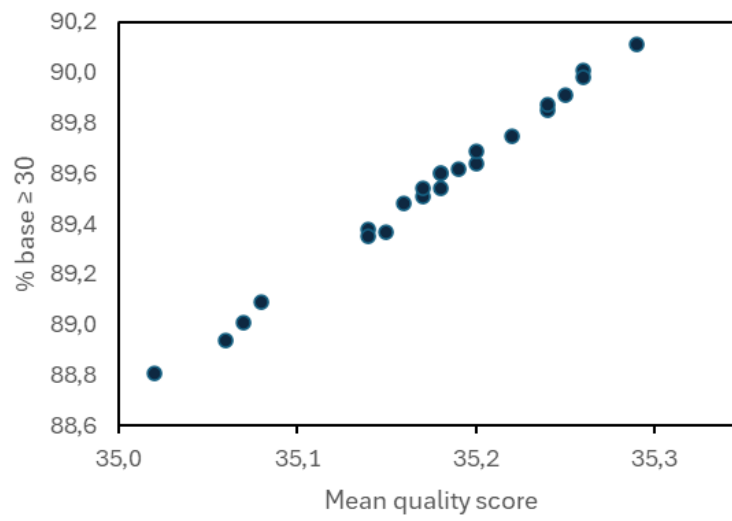

C

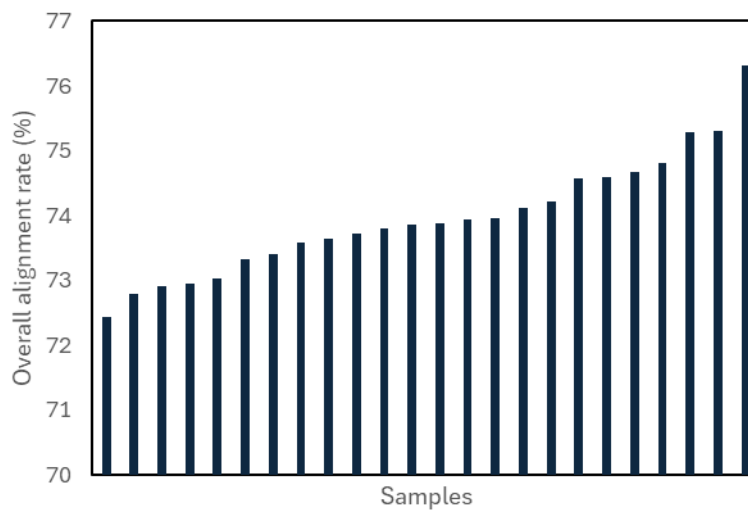

Supplement: Supplementary file 1 — Supplementary Material 1: Supplementary Fig. 1. Total reads number (A), reads quality (B) and overall mapping rate (C) of RNA-seq data. On B, each dot represent a sample. [file 12917_2025_4902_MOESM1_ESM.pdf]
